# Cost-utility analysis of different venous access devices in breast cancer patients: a decision-based analysis model

**DOI:** 10.1186/s12913-023-09517-1

**Published:** 2023-05-16

**Authors:** Na Chen, Qing Yang, Yin Feng Li, Qin Guo, De Yu Huang, Jia Ling Peng

**Affiliations:** 1 School of Nursing, Chengdu Medical College, Chengdu, 610500 China; 2 Nursing Department, Sichuan Clinical Research Center for Cancer, Sichuan Cancer Hospital & Institute, Sichuan Cancer Center, Affiliated Cancer Hospital of University of Electronic Science and Technology of China, Chengdu, 610041 China

**Keywords:** Breast cancer, Cost-utility analysis, Decision tree, Venous access

## Abstract

**Background:**

Venous access devices commonly used in clinical practice for long-term chemotherapy of breast cancer include central venous catheters (CVCs), peripherally inserted central venous catheters (PICCs), and implantable venous access ports (IVAPs). CVCs and PICCs are less costly to place but have a higher complication rate than IVAPs. However, there is a lack of cost-utility comparisons among the three devices. The aim of this study was to assess the cost-effectiveness of three catheters for long-term chemotherapy in breast cancer patients.

**Methods:**

This study used propensity score matching (PSM) to establish a retrospective cohort. Decision tree models were used to compare the cost-effectiveness of three different intravenous lines in breast cancer chemotherapy patients. Cost parameters were derived from data extracted from the outpatient and inpatient charging systems, and total costs included costs of placement, maintenance, extraction, and handling of complications; utility parameters were derived from previous cross-sectional survey results of the research group; and complication rates were derived from breast cancer catheterization patient information as well as follow-up information. Quality-adjusted life years (QALYs) were measured for efficacy outcomes. Incremental cost-effectiveness ratios (ICERs) were used to compare the three strategies. To assess uncertainty in model parameters, sensitivity analyses (univariate sensitivity analysis and probabilistic sensitivity analysis) were performed.

**Results:**

A total of 10,718 patients (3780 after propensity score matching) were included. IVAPs had the smallest cost-utility ratio, and PICCs had the largest cost-utility ratio when left in place for more than 12 months. The incremental cost-utility ratio of PICC to CVC was $2375.08/QALY, IVAP to PICC was $522.01/QALY, and IVAP to CVC was $612.98/QALY. Incremental cost-effectiveness ratios showed that IVAPs were more effective than CVCs and PICCs. Model regression analysis showed that the IVAP was recommended as the best regimen regardless of the catheter indwelling time (6 months, 12 months or more than 12 months). The reliability and stability of the model were verified by single-factor sensitivity analysis and Monte Carlo simulation (probabilistic sensitivity analysis).

**Conclusion:**

This study provides economic evidence for the selection of vascular access in breast cancer chemotherapy patients. In the case of limited resources in China, establishing a decision tree model comparing the cost-effectiveness of three vascular access devices for breast cancer chemotherapy patients determined that the IVAP was the most cost-effective regimen.

**Supplementary Information:**

The online version contains supplementary material available at 10.1186/s12913-023-09517-1.

## Background

Breast cancer is the most common malignancy in women, causing the largest number of cancer-related deaths [1, 2]. Chemotherapy is one of the main treatments for breast cancer. Systemic intravenous chemotherapy has been recommended for many patients with invasive breast cancer to reduce the risk of recurrence and improve patient outcomes [3, 4]. Some breast cancer patients must receive chemotherapy for more than 6 months [5]. It is well known that chemotherapy administered via peripheral veins is dangerous because of serious side effects, such as extravasation of chemotherapeutic agents, unacceptable pain, and psychological trauma [6, 7]. Ensuring safe infusion of chemotherapy drugs is very important for breast cancer treatment.

Reliable and safe long-term venous access is ideal for cancer patients receiving chemotherapy. Various venous access devices (VADs) have been developed in clinical practice, including peripheral intravenous catheters (PICVs), midline catheters, central venous catheters (CVCs), peripherally inserted central catheters (PICCs), and implantable venous access ports (IVAPs) [8], and systemic chemotherapy can be administered through these devices. Central venous catheters (CVCs), peripherally inserted central catheters (PICCs), and totally implantable thoracic access ports (IVAPs) are commonly used for this purpose [9, 10]. Several systematic reviews have shown [11, 12] that PICCs are associated with more complications during maintenance but fewer complications during catheterization than CVCs and IVAPs [13]. PICCs are rated with more catheter indwelling days than CVCs and PIVCs but fewer than IVAPs [12, 13]. Placement and removal of IVAPs must be performed by a physician in the operating room (OR) or interventional radiology (IR) room, whereas PICCs are usually inserted by professional nurses and do not require OR and IR rooms. Despite the lack of controlled trials, the use of PICCs in cancer patients has increased. Compared with IVAPs, PICCs are associated with more adverse events (deep vein thrombosis, line occlusion, infection, and mechanical events) [9]. While most existing studies focus on cost comparisons of PICCs and other VADs, some researchers have observed that the insertion cost of PICCs is higher than that of PIVCs but lower than that of CVCs and IVAPs [14–16]. Despite the widespread use of systemic chemotherapy in breast cancer patients, the optimal choice of vascular access is unknown [11]. In summary, increased complications impact the quality of life of patients, and the indwelling time varies with type of catheter access; although IVAP has few complications, it is costly. Balancing cost with indwelling time and quality of life is a problem faced by policymakers.

CVC, PICC and IVAP can be used for breast cancer patients with medium- and long-term chemotherapy, but each has advantages and disadvantages. PICCs are rated with more catheter indwelling days than CVCs but fewer than IVAPs [12, 13]. CVC has the problem of repeated catheterization throughout chemotherapy but is associated with reduced maintenance costs and complications during indwelling [11, 12]. At present, the number of studies on the economic evaluation of CVC, PICC and IVAP is limited, and the cost structure is not comprehensive. Most of the studies compare the two infusion pathways, but there is no comprehensive comparison of IVAP, PICC and CVC. The evaluation method is single, the evaluation content is not standard enough, and the quality of the evaluation methodology is defective, which leads to the great inconsistency of the evaluation results. Some researchers have highlighted the extreme lack of economic evaluation studies of peripherally inserted central catheters and other venous accesses in terms of number, evaluation content, and economic evaluation methods [17]. Therefore, it is necessary to conduct a higher quality economic evaluation study of peripherally inserted central catheters and other VADs by establishing relevant models for health economic evaluation. The resulting economic-based evidence will help clinicians, patients, and policymakers select appropriate VADs in clinical scenarios [17]. The hypothesis of this study is that IVAP is the most cost-effective solution at 6 months, 12 months, and beyond 12 months. Meanwhile, the market price of catheters decreases with the extension of time, which will further reduce the cost of IVAP, thus making the cost-effectiveness of IVAP more important. The purpose of this study is to confirm whether the above hypothesis is true. Based on this, it may provide reliable evidence for the clinical selection of vascular access with higher cost and effectiveness and provide a basis for the formulation of health care policy.

## Methods

This study was designed to compare the cost and efficacy of CVC, PICC and IVAP vascular accesses from catheter placement to removal for long-term chemotherapy in breast cancer patients from a medical institution perspective. A decision analytic model was developed to perform a cost-utility analysis to assess the economic impact of the three vascular accesses. The incremental cost-effectiveness ratio (ICER) is defined as the cost per QALY gained. The model adopts a decision tree structure, as shown in Supplementary Material 2, Fig. S1. The study was designed as a retrospective cohort study and included additional cross-sectional surveys (utility values for each vascular access were derived from previous cross-sectional surveys using the EQ-5D-5 L health scale as a measurement tool). In the retrospective cohort study, propensity score matching (PSM) was used to reduce selection bias and balance the baseline characteristics of the three groups. Ethical approval for the study was obtained from the Ethics Committee of the research hospital (approval number SCCHEC-02-2021-053). Studies were reported according to the Uniform Standards Statement for Reporting Health Economic Evaluations [18].

### Model structure

We constructed a decision tree model in which CVC, PICC, and IVAP were the three main branches of the decision tree; CVC had 10 subbranches, PICC had 9 subbranches, and IVAP had 8 subbranches. There were 27 possible paths (left to right) at the start of the study. The three pathways were divided into those with and without complications, and the type of each vascular access complication was included in the model as a constituent ratio. The decision analytic model identified probability-, cost-, and quality-adjusted life years for three different venous access lines. The structure of this model is shown in Supplementary Material 2, Fig. S1.

### Setting and operating procedures

This study was conducted in a tertiary grade A cancer hospital in Chengdu, China. Because this hospital is one of the training bases for static therapy specialist nurses in China, the operations carried out in this hospital are standardized and representative. Because it is one of the first hospitals to establish vascular access clinics in China and the daily number of visits is large, it has a wealth of CVC, PICC and IVAP data. In the hospital, catheter placement, maintenance and removal were performed according to standard operating procedures based on the first national industry standard for intravenous infusion in China issued and implemented in 2014, “Technical Operating Procedures for Intravenous Infusion Care”, “Practice Standards for Infusion Therapy in 2016” [8] and “Clinical Guidelines for Venous Access in Oncology” [19]. CVC and PICC placement was performed under ultrasound guidance in the catheterization laboratory by trained and qualified nurses. The CVC was removed immediately after the completion of each chemotherapy cycle. There was an intermittent period between two catheterization procedures, and then the CVC was reinserted during the next chemotherapy cycle. CVCs used German Braun V330 and Dior 6Fr catheters. The PICC was a 4Fr high-pressure-resistant single-lumen polyurethane catheter and a three-way valve catheter manufactured by Bard. Usually, the CVC approach involves the internal jugular vein and femoral vein, and the PICC approach involves the brachial vein, basilic vein, median cubital vein and cephalic vein. The tip of the CVC and the PICC in the internal jugular vein were placed at the cardiocaval junction (CAJ), and the tip of the CVC in the femoral vein was located at the level of the transverse septum. Localization examination was performed by chest X-ray. CVC and PICC were maintained and removed weekly by vascular access outpatient or inpatient ward nurses, who performed flushing, sealing with saline and sodium heparin lock solution and changing of patch dressings. IVAP placement was performed by surgeons and catheter lab nurses using ultrasound guidance in the outpatient operating theatre; the IVAPs in this study were all chest wall ports. The IVAP was a Braun’s 04438663 catheter and BD’s 7Fr three-way valve type single-lumen catheter. The approach for IVAP was the internal jugular and axillary veins, and intraoperative localization was performed by the EKG localization technique with the catheter tip located at the cardiocaval junction (CAJ). IVAP was maintained monthly by vascular access outpatient or inpatient ward nurses with heparinized saline flushes and dressing changes. The IVAP was removed with the assistance of a doctor and nurse in the outpatient operating room, and the wound was covered with gauze and dressing.

### Participants

This retrospective cohort study included all patients who required medium- and long-term chemotherapy via CVC, PICC, or IVAP and completed the entire catheterization to removal process in the vascular access information system of the study hospital from January 2016 to December 2020. The inclusion criteria were as follows: (1) age greater than 18 years; (2) need for chemotherapy via CVC, PICC, or IVAP; (3) first insertion of CVC, PICC, or IVAP; (4) catheter placement, maintenance, and removal performed in this hospital; and (5) catheter maintenance performed according to standard operating procedures except in cases of complications. The exclusion criteria were as follows: (1) contraindications for VADs other than IVAP (i.e., patients after bilateral radical mastectomy); and (2) incomplete placement, maintenance, and extraction information. For the cross-sectional survey, the criteria for patient inclusion were as follows: (1) oncologic patients who underwent central venous catheterization and were in the maintenance period with the catheter; (2) patients 18 years of age and older; and (3) patients who did not have any mental health problems, were expressive, and had no obstacles to communication. In addition, the patients agreed to participate in this study. Informed consent was obtained from all participants. We excluded patients who were catheterized but prepared for extubation.

### Cost calculation

From a medical institution perspective, only direct medical costs were considered. Total costs from catheter insertion to removal included costs of placement, maintenance, removal, and management of complications. The total maintenance cost was calculated by multiplying the cost of a single maintenance visit and the number of maintenance visits. The cost of complications included the cost of treating all complications from insertion to removal, calculated as the sum of the costs of registration, laboratory tests (e.g., routine blood and blood culture), imaging tests (e.g., ultrasound, chest X-ray, and venography), drugs, materials, and treatment. All costs were calculated in RMB and converted to USD at the standard exchange rate of 1 USD = 6.9838 RMB (17 Sep 2022).

### Health outcomes

Health outcome measures were expressed as quality-adjusted life years, which were calculated as median survival time * utility value. Secondary outcomes were complication rates and included all complications from insertion to removal, including catheter slippage, puncture site oozing/exudation, CRBSI, local infection, catheter rupture, drug extravasation, skin hypersensitivity, skin injury, venous thrombosis, and catheter occlusion.

### Cost-utility analysis

In this study, we used the cost-utility ratio (CER) to compare the cost-effectiveness of CVC, PICC and IVAP. In addition, the incremental cost-effectiveness ratio (ICER) is a complementary result reflecting the impact of willingness to pay on the choice of venous access if costs and utilities meet the criteria of Cost 1 > Cost 2, Utility 1 > Utility 2, and CER 1 > CER 2. The lower the CER is, the higher the cost utility of the vascular access. In addition, vascular access is an acceptable option according to cost utility if the ICER falls within the desired value of the outcome. The formula is as follows [20]: CER = Cost/Effectiveness; ICER = Cost_1_ - Cost_2_/Effectiveness_1_ - Effectiveness_2_. In the formula, subscripts 1 and 2 refer to the calculated PICC and CVC; IVAP and PICC; or IVAP and CVC values, respectively. If the cost of a PICC is lower than that of an IVAP and the efficacy is higher than that of an IVAP, the PICC is clearly superior, so ICER calculation is not necessary. Conversely, if IVAP costs and utilities are both higher than those of CVC and PICC, incremental cost-effectiveness ratios need to be calculated and compared to willingness to pay. For all patients, all calculations were performed twice.

### Data collection

For the retrospective cohort study, all data (material cost, maintenance time, complications and cost, indwelling time), including general patient information and placement, maintenance, and removal information, were collected from the catheterization and extubation department in the catheterization laboratory of the study hospital from January 2016 to December 2020 and from the electronic information system and inpatient and outpatient charging system in the information department. Cross-sectional surveys were collected from June 2021 to October 2021 for patients undergoing catheter maintenance in vascular access clinics at cancer hospitals and for patients with catheters in the catheterization period among inpatients.

### Statistical analysis

TreeAge Pro 2021 software was used to establish the decision tree model for cost-effectiveness analysis; Excel was used to manage and sort the data; and R software was used for propensity score matching. Stata/SE15.1 software was used to establish the competing-regression risks for survival analysis, determine the median survival time of the three vascular pathways, fit the survival curves, compare the AIC values of each distribution, and select the optimal distribution for each survival curve; other statistical analyses were also performed using Stata/SE15.1 software. Descriptive statistical analyses were performed on demographic characteristics before and after PSM. Continuous data are described as the mean ± standard deviation, and categorical data are described as frequencies and percentages. To reduce selection bias and balance the baseline in the retrospective cohort, PSM methods [21] were used to match study subjects for CVC, PICC, and IVAP. Matching criteria were age, sex, ethnicity, education, height, weight, presence of hypertension, presence of hyperlipidemia, smoking history, allergy history, and thrombosis history. A 1:2 nearest neighbor match between patients with CVCs, PICCs and IVAPs was performed. The caliper value was 0.1. A chi-square test or analysis of variance was used to determine whether the baseline data of patients after PSM were consistent between the CVC, PICC and IVAP groups. A chi-square test was used to compare whether there was a statistically significant difference in the incidence of complications among the three groups. A value of p < 0.05 was considered statistically significant. Missing values were supplemented by consulting medical records and source data to ensure data integrity.

### Sensitivity analysis

We used univariate ascertainment sensitivity analysis and Monte Carlo probabilistic sensitivity analysis (PSA) to assess uncertainty in cost-utility outcomes. In DSA, one input parameter is changed at a time to keep all other parameters at base-case values, and the input parameters use either 95% CIs or a decrease and increase of 10% as the upper and lower limits of their values [22]. In addition to the type of complication, the remaining 37 parameters were included in the univariate sensitivity analysis, and their results were presented as cyclones. In the PSA, we performed 1000 simulations, varying input parameters over a range and applying different distributions. We set the cost parameter to a gamma distribution because of the asymmetric distribution of costs. Because the probability parameter and health utility value are parameters restricted between 0 and 1, we used a beta distribution. For the median survival time, we finally determined the distribution for CVC to be a lognormal distribution and the distribution for PICC and IVAP to be a Gompertz distribution according to the survival curve fitting results (Supplementary Material 1, Table S2). The interval period, mean catheterization times and catheterization intervals of CVC were set as normal distributions because they exhibited a central tendency. Probabilistic sensitivity analysis results were presented as cost-effectiveness acceptability curves and scatter plots. Given the short time frame, neither costs nor utilities were discounted.

## Results

### Study population

Of the 10,718 patients eligible for the retrospective study, we included 3,780 patients after propensity score matching; 1512 patients had CVCs, 756 patients had PICCs, and 1512 patients had IVAPs. All variables had p values greater than 0.05 after matching, meaning the difference was not statistically significant, and the demographic characteristics of the three venous accesses were consistent at baseline. The demographic characteristics of the patients included before and after PSM for the three types of vascular access are presented in Supplementary Material 1, Table S1.

### Complications

The complication rates for the three vascular accesses are shown in Table 1. There were significant differences among the three vascular accesses with and without complications (χ^2^ = 19.748, p < 0.001), and there were significant differences among the types of complications (χ^2^ = 123.990, p < 0.001).

**Table 1:** Complications of CVC,PICC and IVAP

Complications	CVC		PICC		IVAP		Statistic	p value
	n = 111 (%)	n = 1512 (%)	n = 55 (%)	n = 756 (%)	n = 58 (%)	n = 1512 (%)		
Type of complications							χ^2^ = 123.990	p < 0.001
Partial slippage of catheter	13 (11.71)	13(0.86)	7 (12.73)	7 (0.93)	-	-		
Local infection	3 (2.70)	3(0.20)	6 (10.91)	6 (0.79)	10 (17.24)	10(0.66)		
Exudation	27 (24.33)	27(1.79)	14 (25.45)	14 (1.85)	1 (1.72)	1(0.07)		
Catheter occlusion	16 (14.41)	16(1.06)	3 (5.45)	3 (0.40)	34 (58.62)	34(2.25)		
CRBSI	1 (0.90)	1(0.07)	-	-	-	-		
Venous thrombosis	9 (8.11)	9(0.60)	8 (14.55)	8 (1.06)	8 (13.79)	8(0.53)		
Allergy	7 (6.31)	7(0.46)	8 (14.55)	8 (1.06)	1 (1.72)	1(0.07)		
Skin damage	19 (17.12)	19(1.26)	7 (12.73)	7 (0.93)	-	-		
Complete catheter slippage	16 (14.41)	16(1.06)	-	-	-	-		
Catheter rupture	-	-	2 (3.63)	2(0.26)	-	-		
Drug extravasation	-	-	-	-	3 (5.17)	3(0.20)		
No damage needle slippage	-	-	-	-	1 (1.72)	1(0.07)		
Total number of patients with complications	111	111 (7.34)	55	55 (7.28)	58	58 (3.84)	χ^2^ = 19.748	p < 0.001

CRBSI: catheter-related bloodstream infection

### Cost

Table 2 summarizes the parameters used for model input, where costs include the costs of catheterization, maintenance, and removal of the three vascular access devices as well as the costs of handling different complications. The base-case analysis results of this study are shown in Table 3, with a total cost of $542.36 for CVC, $827.82 for PICC, and $2043.12 for IVAP.

**Table 2:** Input data values for base case, one-way sensitivity analysis and probabilistic sensitivity analysis

Parameter	Mean ( SD)	95%(Confidence Interval )	Distribution
c1_CVC_insertion	68.0747 (43.2521)	(66.7348,69.4147)	Gamma
c1_CVC_maintenance	41.9615 (45.6716)	(40.4214,43.5015)	Gamma
c1_CVC_removal	8.2753 (3.3928)	(8.1466,8.4041)	Gamma
c2_PICC_insertion	349.5522 (102.7811)	(329.5638,369.5405)	Gamma
c2_PICC_perMaintenance	21.6773	(0.433,42.9217)	Gamma
c2_PICC_removal	7.2909 (3.4561)	(6.7812,7.8007)	Gamma
c3_IVAP_insertion	844.8203 (317.5976)	(828.9301,860.7106)	Gamma
c3_IVAP_perMaintenance	32.1395	(0.6413,63.6376)	Gamma
c3_IVAP_removal	82.3272 (6.8959)	(81.5174,83.137)	Gamma
c_complication_allergy	30.7898 (52.408)	(-13.0244,74.604)	Gamma
c_complication_CatheterRupture	44.1021	(35.2817,52.9225)	Gamma
c_complication_CRBSI	47.1949	(37.7559,56.6339)	Gamma
c_complication_drugExtravasation	25.5226 (0.3675)	(22.2205,28.8248)	Gamma
c_complication_exudation	28.8435 (23.8289)	(11.7973,45.8896)	Gamma
c_complication_infection	131.2002 (196.0126)	(-50.0812,312.4816)	Gamma
c_complication_Nslippage	2.2624	(1.8099,2.7149)	Gamma
c_complication_occlusion	38.6511 (36.5992)	(19.8335,57.4687)	Gamma
c_complication_Pslippage	152.7048 (61.313)	(0.3948,305.0149)	Gamma
c_complication_skinDamage	14.2501 (3.7543)	(-19.4812,47.9815)	Gamma
c_complication_thrombosis	17.0416 (19.8661)	(-3.8066,37.8898)	Gamma
c_complication_Tslippage	3.8460 (2.1042)	(-1.381,9.0731)	Gamma
CVC_intermission	0.0686 (0.1772)year	(0.06174,0.07546)	Normal
CVC_mean_insertionTimes	4.5589 (2.8271)	(4.416248,4.701477)	Normal
CVC intervals	3.5589 (2.8271)	(3.416348,3.701477)	Normal
p1_CVC_complication	0.0734	-	Beta
p2_PICC_complication	0.0728	-	Beta
p3_IVAP_complication	0.0384	-	Beta
T1_CVC_medianSurvivalTime	0.01096(year)	-	Lognormal
T2_PICC_maintenanceInterval	0.0192(year)	-	-
T2_PICC_medianSurvivalTime	0.4137(year)	-	Gompertz
T3_IVAP_maintenanceInterval	0.0822(year)	-	-
T3_IVAP_medianSurvivalTime	2.8493(year)	-	Gompertz
u1_CVC	0.8989706 (0.0644212)	(0.8753407,0.9226005)	Beta
u1_CVC_complication	0.8778395 (0.0228473)	-	Beta
u1_CVC_intermission	0.814[32]	-	Beta
u2_PICC	0.8806194 (0.0565468)	(0.8602321,0.9010067)	Beta
u2_PICC_complication	0.862279 (0.0614081)	(0.8054858,0.9190719)	Beta
u3_IVAP	0.9448492 (0.0726747)	(0.9340994,0.955599)	Beta
u3_IVAP_complication	0.942318	-	Beta

### Health outcomes

The health parameters used for model input are shown in Table 2. In our model, the health utility values of CVC, PICC, and IVAP were 0.8989706, 0.8806194, and 0.9448492, respectively; their median survival times were 0.01096 years, 0.4137 years, and 2.8493 years, respectively, and their survival curves are shown in Supplementary Material 2, Fig. S2. The quality-adjusted life years were 0.24 years for CVC, 0.36 years for PICC, and 2.69 years for IVAP (Table 3); IVAP had the longest QALYs.

Because CVC involves multiple catheterizations throughout the treatment cycle, the quality-adjusted life years for CVC are composed of two parts: QALYs during the intervals between catheterizations and QALYs during the intervals with catheterization (involving the average number of catheterizations, length of intervals, and number of intervals between catheterizations). This outcome represents quality-adjusted life years for patients to complete the entire treatment cycle using CVC rather than quality-adjusted life years with a single catheterization. Table 2 shows that the median survival time of CVC is the median survival time of catheterization.

### Cost-utility analysis

The results of the cost-utility analysis for the three vascular accesses are presented in Table 3 and Supplementary Material 2, Fig S3. Table 3 presents the results of the scenario analysis, which are summarized as follows: (1) The cost-effectiveness ratios of CVC and PICC did not change with indwelling catheter times of 6 months, 12 months, and more than 12 months, while the cost-effectiveness ratios of IVAP decreased over time. IVAP had the smallest cost-utility ratio, and PICC had the largest cost-utility ratio after catheter indwelling for more than 12 months. However, the cost-utility ratio is not enough to conclude that IVAP is the optimal scheme. (2) In the incremental cost-utility analysis, when the catheter indwelling time was 6 months, the incremental cost-utility ratios of IVAP to CVC and PICC were greater than that of PICC to CVC; when the indwelling time was 12 months, the incremental cost-utility ratios of IVAP to CVC and PICC were smaller than that of PICC to CVC; and when the indwelling time was more than 12 months, the incremental cost-utility ratio of PICC to CVC was $2375.08/QALY, IVAP to PICC was $522.01/QALY, and IVAP to CVC was $612.98/QALY. Regardless of the length of dwell time, IVAP had the best cost-utility regimen based on the incremental cost-utility ratios and compared to WTP.

**Table 3:** Cost-effectiveness analysis for CVC, PICC and IVAP

	6m DT				12m DT				More than 12m DT			
	Cost^a^($)	QALY	CER	ICER	Cost($)	QALY	CER	ICER	Cost($)	QALY	CER	ICER
CVC	542.36	0.24	2226.71	-	542.36	0.24	2226.71	-	542.36	0.24	2226.71	-
PICC	827.82	0.36	2275.74	^b^2375.08	827.82	0.36	2275.74	^b^2375.08	827.82	0.36	2275.74	^b^2375.08
IVAP	1121.91	0.47	2407.77	^c^2877.76	1320.06	0.94	1397.26	^c^847.24	2043.12	2.69	758.99	^c^522.01
				^b^2606.08				^b^1109.12				^b^612.98

Note. DT:dwell time; m:month; QALY:Quality-Ajusted Life Years; CER:cost-effectiveness ratio; ICER:incremental cost-effectiveness ratio

^a^The average total cost; ^b^CVC; ^c^PICC

### Sensitivity analysis

**Fig. 1:**
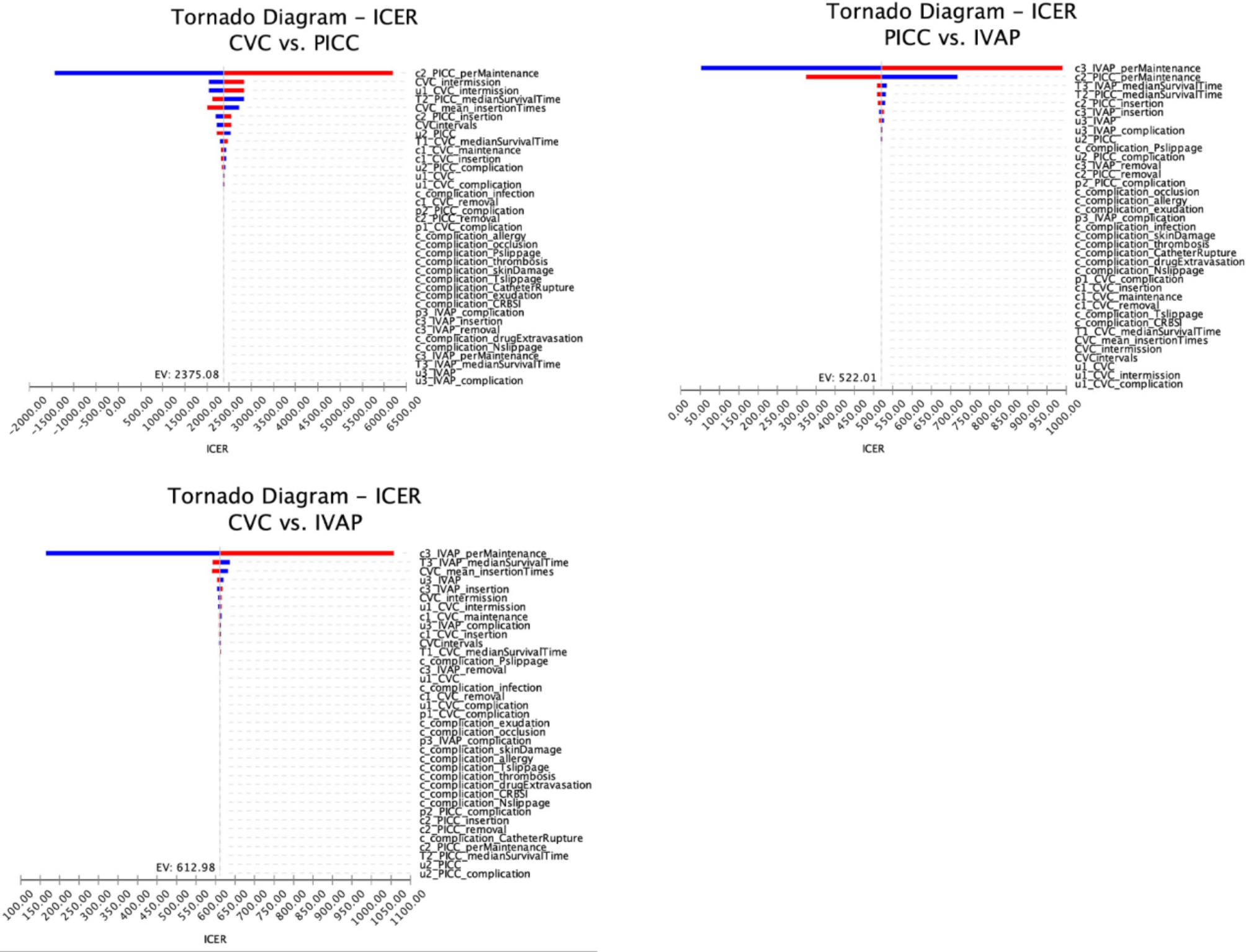
Tornado plot showing one-way sensitivity analysis comparing the three vascular accesses

**Fig. 2:**
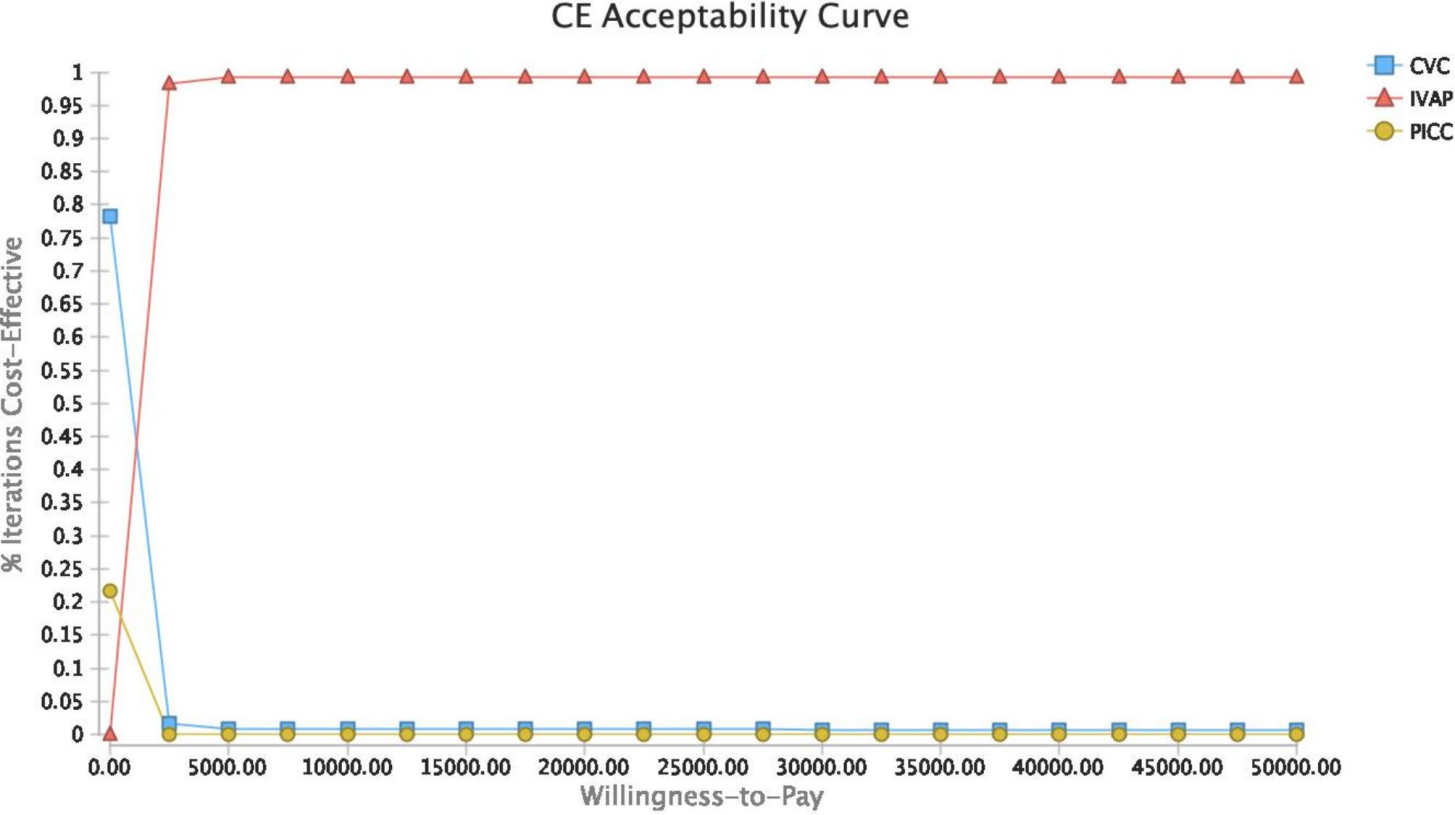
Acceptance curve of probability sensitivity analysis

We used univariate sensitivity analysis, the results of which are shown in Fig. 1 (cyclone plot), and probabilistic sensitivity analysis, the results of which are shown in Fig. 2 and Supplementary Material 2, Fig. S4 and Fig. S5 (acceptable curve, scatter plot, and histogram).

### Univariate sensitivity analysis

We performed univariate sensitivity analyses of cost, utility, and complication rates for each of the three vascular accesses. As shown in the cyclone results in Fig. 1, the single maintenance costs (c2_PICC_perMaintenance, c3_IVAP_perMaintenance) of PICCs and IVAPs had a greater impact on the results. When all parameters changed within the specified ranges, the ICER remained below the WTP value. This result is consistent with the basic analysis results, indicating that the basic analysis results are robust.

### Probabilistic sensitivity analysis

Probabilistic sensitivity analysis was also performed to explore the effect of changes in parameter distributions on the results. It is assumed that the cost parameters of the three vascular accesses obey a gamma distribution; utility parameters and complication rates obey a beta distribution; median survival time for CVC obeys a lognormal distribution, and median survival times for PICC and IVAP obey a Gompertz distribution; and interval period, mean catheterization times, and catheterization intervals of CVC obey a normal distribution. Monte Carlo simulation was used for cost-effectiveness probability sensitivity analysis of each parameter to obtain cost-effectiveness acceptance curves, scatter plots, and histograms as shown in Fig. 2 and Supplementary Material 2, Fig. S4 and Fig. S5, respectively. When the willingness to pay (WTP) per unit QALY is 0, the probability of CVC being the optimal scheme is 100%. With increasing WTP per unit QALY, the probability of CVC being the optimal scheme decreases, while the probabilities of PICC and IVAP being the optimal scheme increase. When the WTP per unit QALY was $3479.48, IVAP exceeded CVC, and the probability of IVAP being the optimal scheme became maximal among the three schemes (Fig. 2). As shown in Supplementary Material 2, Fig. As shown in Figure S4, the cost-utility scatter plot of the three groups showed a central distribution trend, and the cost-utility scatter plot of IVAP was centrally distributed in the upper right part of the CVC and PICC plots, indicating that the cost and utility of IVAP were higher than those of CVC and PICC. With a WTP of $34794.8108/QALY, the probability of selecting IVAP was 95%, the probability of selecting PICC was 4.2%, and the probability of selecting CVC was 0.8% (Supplementary Material 2, Fig. S5).

## Discussion

This study was designed to investigate the cost-effectiveness of three vascular access options by calculating incremental cost-effectiveness ratios and comparing them with willingness to pay to obtain the optimal regimen suitable for breast cancer patients. To our knowledge, this is the first study to (a) evaluate the cost utility of three different vascular accesses—CVC, PICC, and IVAP—in breast cancer chemotherapy patients from a Chinese perspective, (b) use real-world costs and probabilities, and (c) use the EQ-5D-5 L health measurement tool combined with a Chinese health utility scoring system to obtain health utility values for the three vascular accesses. In addition, all data from this study came from actual patients. Our study demonstrates that IVAP is the optimal regimen according to the incremental cost-utility ratio compared to WTP. IVAP was also found to have the lowest complication rate from catheter placement to removal. The results of this study provide economic evidence to help policymakers and clinicians select the most cost-effective vascular access for chemotherapy in breast cancer patients.

To date, studies on the cost-utility status of CVC, PICC, and IVAP have been rather limited [17]. The most frequently reported measure of benefit in economic evaluations is quality-adjusted life years (QALYs) [23, 24], which combines health-related quality of life (HRQoL) and longevity into a single summary measure. The primary outcome measure of this study was the quality-adjusted life years used, and the utility values involved in calculating QALYs were obtained from cross-sectional surveys conducted earlier in this study. Olivia et al. [25] reported an economic evaluation that showed that the mean cost of the Hickman catheter over a 1-year period was significantly higher, fewer complications were observed, and the mean QALYs were lower compared to TIVAS in a base-case analysis. The results of their study also suggested that TIVAS could be a cost-effective option compared to the Hickman catheter. This conclusion is consistent with our findings, but CVC was less costly than IVAP in our study. This difference is due to the high complication rate of the Hickman catheter in the study by Olivia et al. [25]. Duclos et al. [26] found that PICCs had a higher overall cost and a higher patient benefit rate than IVAPs, with an incremental cost-effectiveness ratio of €400.24 at the end of 150 days of chemotherapy. However, they concluded that IVAP was a more cost-effective device. Their conclusions are consistent with our findings, but the total cost and QALYs of PICCs were lower than those of IVAPs in our study. This may be related to the different cost compositions and outcome measures used. Our outcome measure employed quality-adjusted life years, whereas the primary efficacy endpoint of Duclos et al. [26] was the proportion of patients who benefited from the entire chemotherapy regimen at the end of the time frame (150 days postexposure). Wang Kairong et al. [27] showed similar results between September and December. The researchers noted [27] that PICCs were more cost-effective than IVAPs for indwelling times of 3–9 months; however, IVAPs may be more cost-effective than PICCs for indwelling times of 9–12 months. The study [27] compared the cost-effectiveness of PICCs and IVAPs with tubes in subgroups, while our study compared the cost-effectiveness based on the median survival time from catheterization to removal of CVCs, PICCs and IVAPs. At the same time, we also performed scenario analysis. The final result is that whether the indwelling time is 6 months, 12 months or more than 12 months, the regression analysis model results are in favor of IVAP as the optimal program. Although the single maintenance costs of PICCs and IVAPs had the largest impact on the model in the univariate sensitivity analysis, the model remained stable when all parameters were varied within the specified range. As shown in Table 3, the acceptance rate of IVAP increases as the indwelling time increases.

In addition, several cost analyses have been performed to compare the total cost of PICCs and IVAPs from catheter insertion to removal [17]. For the comparative results of costs, our study had findings consistent with those of the study by Fang et al. [28], which involved similar health systems, cost composition, and material prices to our study. Our results differs from those of Taxbro et al. [9] and Patel et al. [29]. These differences were due to the higher cost of PICC placement than IVAP in the study by Taxbro et al. [9]; this variation relative to our study may reflect different types and prices of catheters in different countries and regions. The inconsistent cost comparison in the study by Patel et al. [29] may be because the PICC was inserted by a radiologist; the material price and cost composition were also different, and the cost of removal was not included. Researchers in various studies [30] have compared the complication rate and cost of the Hickman tunneled catheter (Hickman), peripherally inserted central catheter (PICC) and totally implanted port (PORT) in patients undergoing systemic anticancer therapy. Their findings indicate that PORT is superior to the Hickman catheter and PICC, and PORT is recommended for patients undergoing systemic anticancer therapy for solid tumors.

Comparing the median survival time and complication rate by catheters, one study [29] revealed that the complication rate of IVAPs was significantly lower than that of PICCs, and the median survival time of PICCs was shorter than that of IVAPs. This is consistent with the results of our study, although our study differs from these studies in that we followed up through catheter survival—not only for 1 year of indwelling time—and analyzed it using competing risk models. In recent years, there have been health economic evaluation studies using competing risk models for survival analysis [31]. This is also a strength of this study.

Our study showed that although IVAP is more costly than CVC and PICC, it has a lower complication rate, a higher utility value, and longer QALYs than CVC and PICC. From the results of this study, the probability of choosing IVAP is as high as 95% at the WTP threshold in China. Over time, the market price of catheters has declined, which will further reduce the cost of IVAPs and thus make the cost utility of IVAPs more substantial.

### Study limitations

Because of the relationship between resources and time, this study was completed in a single center. Our cost data are retrospective and include only direct health care costs; the use of this result in the health system may be limited. A prospective study could be used in the future to perform a multicenter cost-utility analysis in different countries and regions. Another limitation of our study is the inability to determine the stage of tumor disease and the effect of treatment choice on the choice of infusion mode. In future studies, we will consider how these factors influence vascular access selection.

## Conclusion

Overall, this study is the first to use a decision tree model to perform a cost-utility comparison of three different vascular accesses—CVC, PICC, and IVAP—in breast cancer chemotherapy patients. The results showed that IVAP was more cost-effective than CVC and PICC from the perspective of Chinese medical institutions. Our analysis will help clinicians make the best decisions when performing vascular access selection in breast cancer chemotherapy patients.

## Electronic supplementary material

Below is the link to the electronic supplementary material.

Supplementary Material 1

Supplementary Material 2

## Data Availability

The dataset are available from the corresponding author on reasonable request.

## Abbreviations

QALY: Quality-adjusted life year
CVC: Chemotherapeutic central venous catheters
PICC: Peripherally inserted central catheter
IVAP: Implantable venous access port
VAD: Various venous access devices
PIVC: Peripheral intravenous catheters
OR: Operating room
IR: Interventional radiology
CER: Cost-effectiveness ratio
ICER: Incremental cost-effectiveness ratio
PSM: Propensity score matching
CAJ: Cardiocaval junction
CRBSI: Catheter-related bloodstream infection
DSA: Deterministic sensitivity analysis
PSA: Probabilistic sensitivity analysis
CI: Confidence intervals
WTP: Willingness to pay
