# Supplementary material for: Cost-utility analysis of different venous access devices in breast cancer patients: a decision-based analysis model
Source: BMC Health Serv Res. 2023 May 16;23:497. doi: 10.1186/s12913-023-09517-1 (PMC10190063; doi:10.1186/s12913-023-09517-1)
Supplement: Supplementary file 1 — Supplementary Material 1 [file 12913_2023_9517_MOESM1_ESM.docx]

Table S1 Demographic characteristics before and after propensity score matching

| Variable | Unmatched | | | | | Matched | | | | |
| --- | --- | --- | --- | --- | --- | --- | --- | --- | --- | --- |
|  | CVC | PICC | IVAP | Statistic | p value | CVC | PICC | IVAP | Statistic | p value |
|  | n=8092(%) | n=918(%) | n=1708(%) |  |  | n=1512(%) | n=756 (%) | n=1512 (%) |  |  |
| Age | 51.29$\pm$9.36 | 49.54$\pm$9.51 | 50.14$\pm$9.23 | F=22.08 | p=0.000 | 50.18 $\pm$9.11 | 49.76$\pm$9.41 | 50.00$\pm$9.51 | F=0.51 | p=0.599 |
| Gender |  |  |  | χ^2^=4.0012 | p=0.135 |  |  |  | - | - |
| Female | 8008(98.96) | 910(99.13) | 1699(99.47) |  |  | 1512(100) | 756(100) | 1512(100) |  |  |
| Male | 84(1.04) | 8(0.87) | 9(0.53) |  |  | - | - | - |  |  |
| Education |  |  |  | χ^2^=74.4698 | p=0.000 |  |  |  | χ^2^=11.8002 | p=0.067 |
| Primary school and below | 2921(36.10) | 297(32.35) | 520(30.44) |  |  | 468 (30.95) | 254 (33.60) | 506 (33.47) |  |  |
| Junior high school | 2984(36.88) | 332(36.17) | 561(32.85) |  |  | 506 (33.47) | 266 (35.19) | 552 (36.51) |  |  |
| High school or technical secondary school | 1217(15.04) | 151(16.45) | 320(18.74) |  |  | 280 (18.52) | 123 (16.27) | 244 (16.14) |  |  |
| College and above | 970(11.99) | 138(15.03) | 307(17.97) |  |  | 258 (17.06) | 113 (14.95) | 210 (13.89) |  |  |
| Nation |  |  |  | χ^2^=0.2003 | p=0.905 |  |  |  | χ^2^=0.000 | p=1.000 |
| Han | 8071(99.74) | 915(99.67) | 1704(99.77) |  |  | 1508 (99.74) | 754 (99.74) | 1508 (99.74) |  |  |
| Other | 21(0.26) | 3(0.33) | 4(0.23) |  |  | 4 (0.26) | 2 (0.26) | 4 (0.26) |  |  |
| Height | 155.69$\pm$5.66 | 156.30$\pm$5.60 | 156.70$\pm$5.47 | F=25.33 | p=0.000 | 156.63$\pm$5.40 | 156.24$\pm$5.54 | 156.25$\pm$5.35 | F=2.27 | p=0.104 |
| Weight | 57.99$\pm$8.71 | 57.14$\pm$8.91 | 57.75$\pm$8.34 | F=4.18 | p=0.0154 | 57.72$\pm$7.94 | 57.30$\pm$8.75 | 57.49$\pm$8.37 | F=0.71 | p=0.492 |
| Hypertension |  |  |  | χ^2^=2.7911 | p=0.248 |  |  |  | χ^2^=0.0038 | p=0.998 |
| No | 7068(87.35) | 812(88.45) | 1514(88.64) |  |  | 1333(88.16) | 666(88.10) | 1332(88.10) |  |  |
| Yes | 1024(12.65) | 106(11.55) | 194(11.36) |  |  | 179(11.84) | 90(11.90) | 180(11.90) |  |  |
| Hyperlipidemia |  |  |  | χ^2^=19.4514 | p=0.000 |  |  |  | χ^2^=1.5657 | p=0.457 |
| No | 7040(87.00) | 790(86.06) | 1417(82.96) |  |  | 1257(83.13) | 640(84.66) | 1280(84.66) |  |  |
| Yes | 1052(13.00) | 128(13.94) | 291(17.04) |  |  | 255(16.87) | 116(15.34) | 232(15.34) |  |  |
| Smoking |  |  |  | χ^2^=5.7210 | p=0.057 |  |  |  | χ^2^=0.8564 | p=0.652 |
| No | 7856(97.08) | 884(96.30) | 1641(96.08) |  |  | 1456(96.30) | 729(96.43) | 1465(96.89) |  |  |
| Yes | 236(2.92) | 34(3.70) | 67(3.92) |  |  | 56(3.70) | 27(3.57) | 47(3.11) |  |  |
| Allergy |  |  |  | χ^2^=0.4795 | p=0.787 |  |  |  | χ^2^=0.1838 | p=0.912 |
| No | 7370(91.08) | 839(91.39) | 1564(91.57) |  |  | 1386(91.67) | 693(91.67) | 1380(91.27) |  |  |
| Yes | 722(8.92) | 79(8.61) | 144(8.43) |  |  | 126(8.33) | 63(8.33) | 132(8.73) |  |  |
| Thrombus |  |  |  | χ^2^=49.0483 | p=0.000 |  |  |  | χ^2^=0.0629 | p=0.969 |
| No | 7810(96.52) | 908(98.91) | 1695(99.24) |  |  | 1503(99.40) | 751(99.34) | 1502(99.34) |  |  |
| Yes | 282(3.48) | 10(1.09) | 13(0.76) |  |  | 9(0.60) | 5(0.66) | 10(0.66) |  |  |

Table S2 Survival Curve Fit Results for Three Venous Access Systems

|  | Exponential | Weibull | Gompertz | Lognormal | Loglogistic |
| --- | --- | --- | --- | --- | --- |
| CVC Survival Curve | | | | | |
| AIC | 21852.37 | 21672.18 | 21037.9 | 19575.39 | 19707.99 |
| PICC Survival Curve | | | | | |
| AIC | 2912.421 | 2866.156 | 2510.343 | 3336.579 | 3286.881 |
| IVAP Survival Curve | | | | | |
| AIC | 3868.354 | 3270.855 | 3098.541 | 4297.024 | 3742.111 |
