# Supplementary material for: Cost-utility analysis of different venous access devices in breast cancer patients: a decision-based analysis model
Source: BMC Health Serv Res. 2023 May 16;23:497. doi: 10.1186/s12913-023-09517-1 (PMC10190063; doi:10.1186/s12913-023-09517-1)

Fig.S1 Decision tree basic model structure


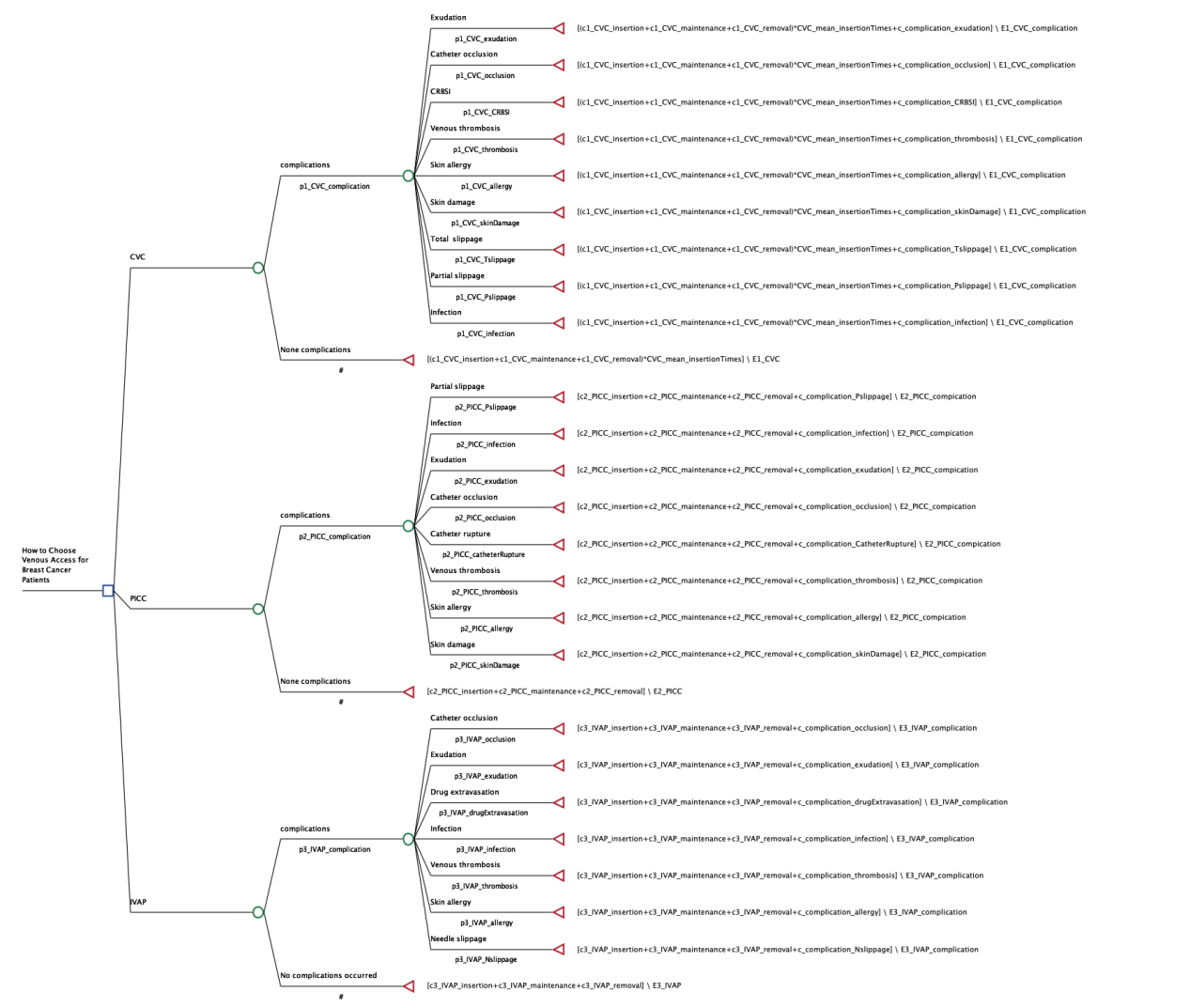


Fig.S2 Survival curves for three vascular accesses


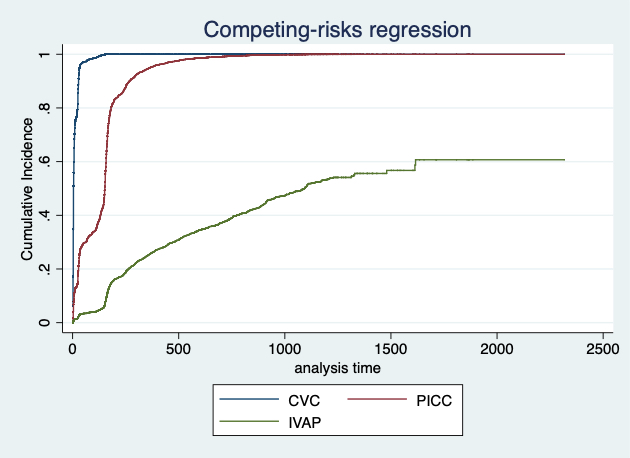


Fig.S3 Cost-effectiveness analysis for CVC、PICC and IVAP


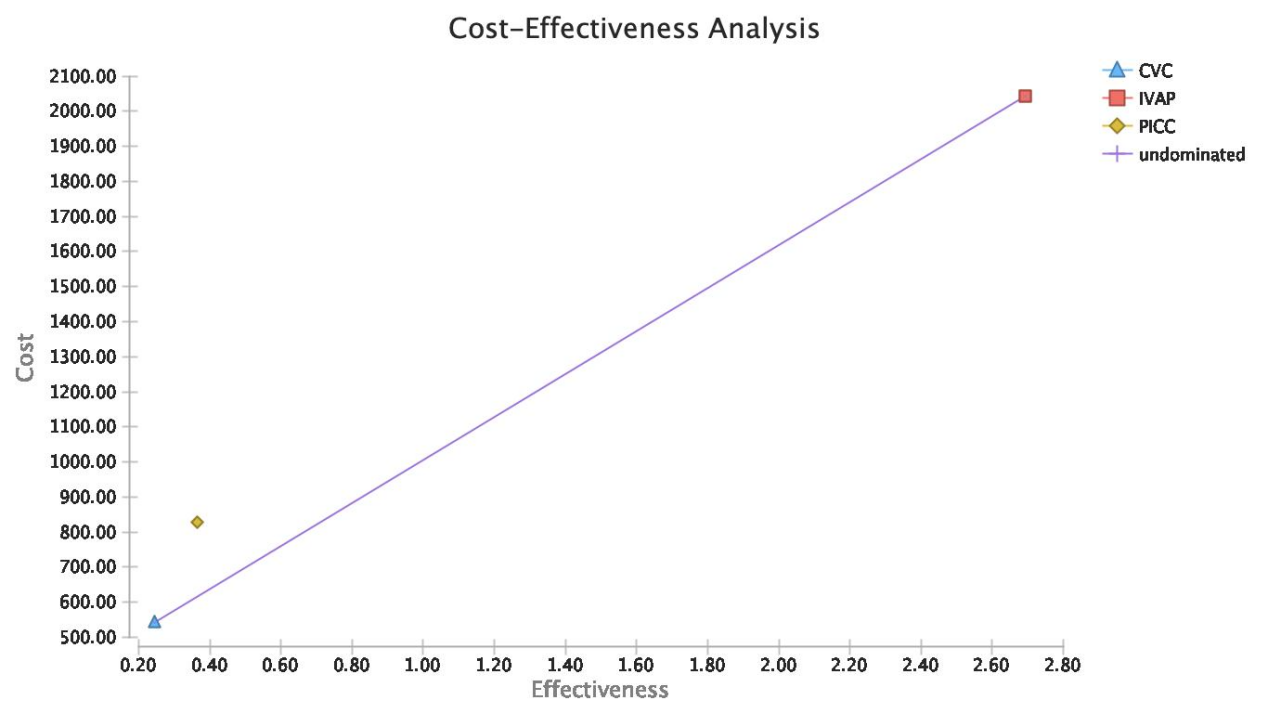


Fig.S4 Scatterplot of probability sensitivity analysis


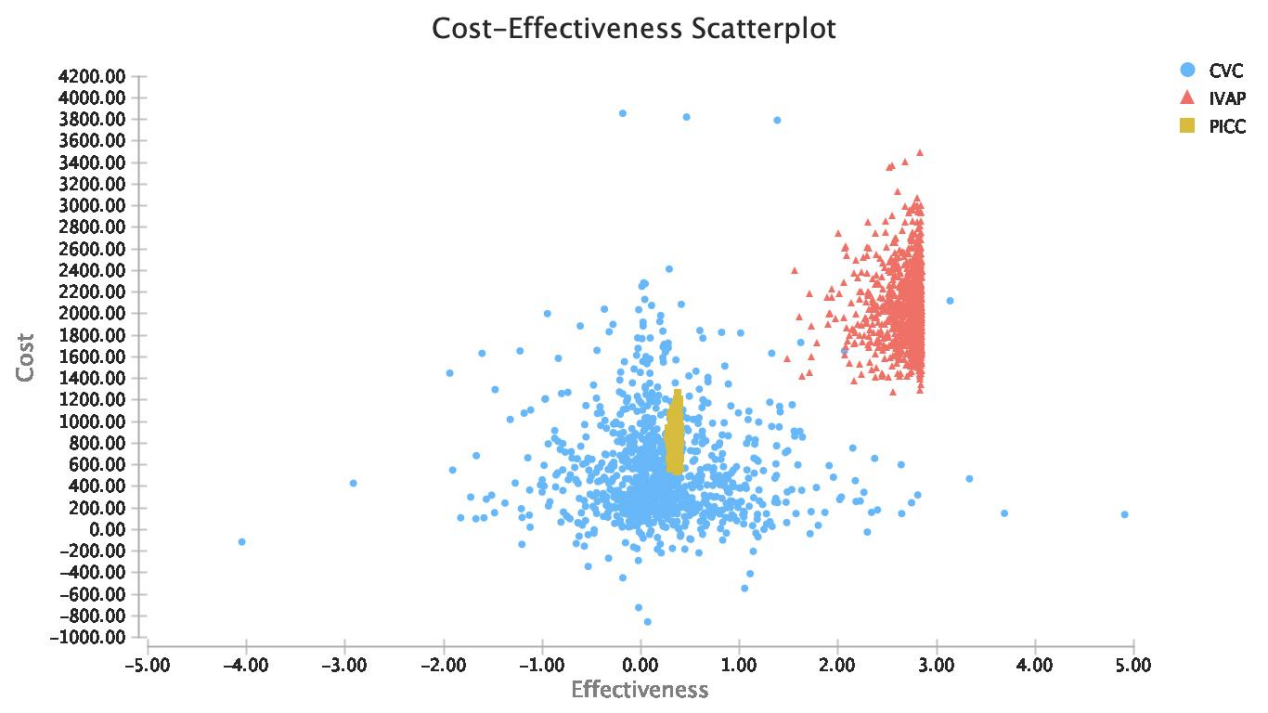


Fig.S5 Histogram of probability sensitivity analysis


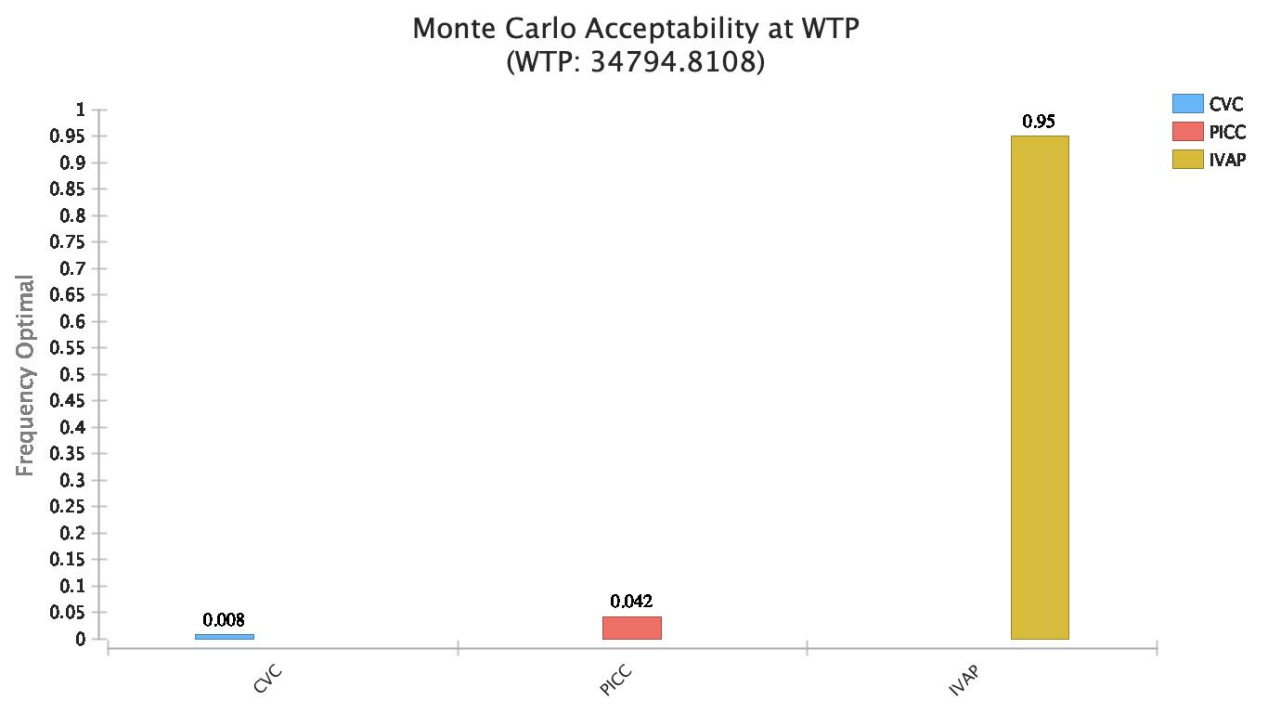

Supplement: Supplementary file 2 — Supplementary Material 2 [file 12913_2023_9517_MOESM2_ESM.docx]
